# Automated time-lapse data segmentation reveals in vivo cell state dynamics

**DOI:** 10.1126/sciadv.adf1814

**Published:** 2023-06-02

**Authors:** Miriam A. Genuth, Yasuhiro Kojima, Dörthe Jülich, Hisanori Kiryu, Scott A. Holley

**Affiliations:** ^1^ Department of Molecular, Cellular and Developmental Biology, Yale University, New Haven, CT, USA.; ^2^ Division of Systems Biology, Graduate School of Medicine, Nagoya University, Nagoya 4668550, Japan.; ^3^ Department of Computational Biology and Medical Sciences, Graduate School of Frontier Sciences, The University of Tokyo, Kashiwa, Chiba, Japan.

## Abstract

Embryonic development proceeds as a series of orderly cell state transitions built upon noisy molecular processes. We defined gene expression and cell motion states using single-cell RNA sequencing data and in vivo time-lapse cell tracking data of the zebrafish tailbud. We performed a parallel identification of these states using dimensional reduction methods and a change point detection algorithm. Both types of cell states were quantitatively mapped onto embryos, and we used the cell motion states to study the dynamics of biological state transitions over time. The time average pattern of cell motion states is reproducible among embryos. However, individual embryos exhibit transient deviations from the time average forming left-right asymmetries in collective cell motion. Thus, the reproducible pattern of cell states and bilateral symmetry arise from temporal averaging. In addition, collective cell behavior can be a source of asymmetry rather than a buffer against noisy individual cell behavior.

## Teaser

Embryonic cell state patterns are noisy but reproducible, and major pattern deviations are due to collective cell behavior.

## INTRODUCTION

Aristotle first noted the astonishing reproducibility of embryogenesis in “*Historia Animalium*,” where he observed, “Generation from the egg occurs in an identical manner in all birds.” For example, in bilaterians, the left and right sides generally adopt an identical form. At the cellular level, development entails a reproducible series of cell state transitions representing changes in gene expression state, physical state, and cell fate. These processes can be noisy; for example, cell migration can be either ordered or disordered, and such disorder is part of normal orderly development (*1*). We now appreciate that gene networks control cell state transitions, but these networks are composed of stochastic molecular processes (*2*). Despite the remarkable progress in the field of developmental biology in recent decades, it has been difficult to study the reproducibility and robustness of cell state transitions in vivo because of the challenge of systematically defining cell states in space and time. Here, we analyze the spatiotemporal pattern of cell state transitions in the zebrafish tailbud.

The vertebrate anterior-posterior body axis develops continuously from head to tail. Progenitors of the trunk mesoderm and spinal cord are largely specified during gastrulation, whereas the tailbud contains a bipotential neuromesodermal progenitor (NMP) cell population that contributes to the posterior body axis (*3*, *4*). In zebrafish, fate mapping experiments indicate that the presomitic mesoderm (PSM) formed during gastrulation gives rise to the first 10 to 13 somites (*5*–*7*). During early somitogenesis, the mesodermal cells continue to join the PSM via medial convergence, and because of their long transit time through the mesodermal progenitor zone (PZ) and PSM, NMP-derived PSM cells primarily contribute to the tail somites. Neighboring NMP cells disperse to contribute to multiple somites along the tail (*8*, *9*).

**Fig. 1.**
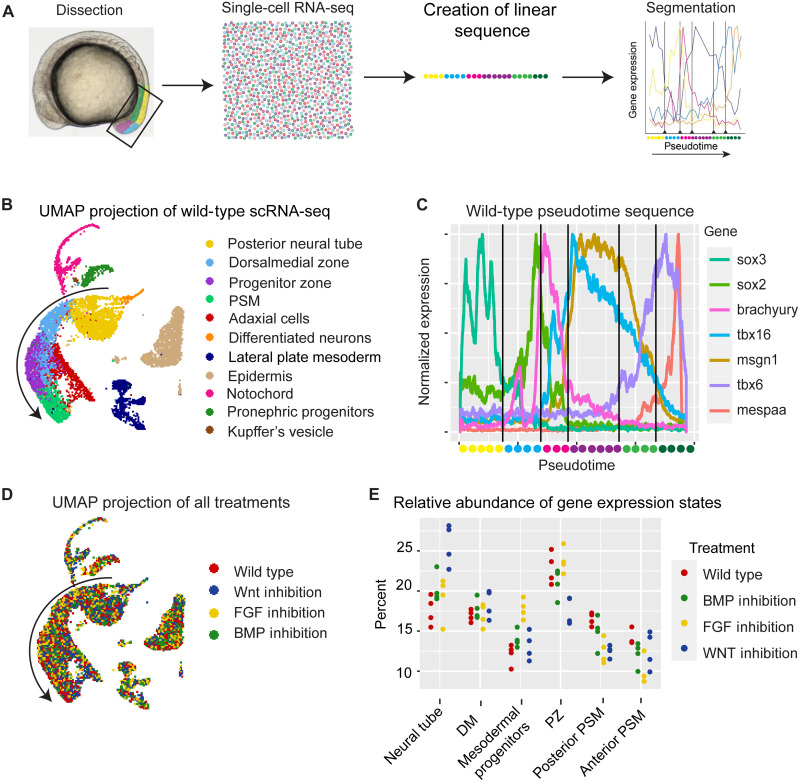
Gene expression cell states in the zebrafish tailbud. (**A**) Schematic of the experimental approach. Tailbuds were dissected and pooled; scRNA-seq profiles were generated, and a one-dimensional pseudotime was created and segmented into gene expression cell states. (**B**) UMAP projection of scRNA-seq data colored by cell type. Arrow marks the path of pseudotime in (C). (**C**) Expression of selected markers over pseudotime. Vertical lines are transition points between cell states as defined by a Bayesian algorithm that minimizes within state statistical error. Note that the segment colors along the pseudotime axis correspond to the colors along the developmental trajectory (arrow) in (B) but with the PZ and PSM being further subdivided into two similarly colored segments in (C). (**D**) UMAP projection of scRNA-seq data colored by experimental treatment. (**E**) Quantification of the differences in the proportion of cells that are in a given cell state in each replicate of each experimental condition. See also figs. S1 and S2.

Cells in the tailbud undergo a series of transitions in gene expression and migratory behavior during their differentiation (Fig. 1A, left) (*9*–*12*). The dorsal-medial (DM) tailbud contains the *sox2/brachyury* expressing NMPs that contribute to both the spinal cord (Fig. 1A, yellow) and the PSM (*3*). In the zebrafish, cells in the DM migrate toward the posterior in a rapid orderly fashion (Fig. 1A, cyan) (*8*, *9*, *13*). At the tip of the tailbud, mesodermally fated DM cells down-regulate *sox2*, up-regulate mesodermal genes such as *tbx16*, and undergo an epithelial-mesenchymal transition (EMT) to migrate ventrally into the PZ (Fig. 1A, magenta). Cell movements in the PZ are more disorderly than the DM. Ultimately, cells leave the PZ, reduce their speed, and assimilate into the left and right PSM (Fig. 1A, green) (*9*, *14*–*16*). Cells in the PSM down-regulate *tbx16* and begin to express *tbx6*. Cell velocity in the anterior PSM declines further as the tissue decreases its volume and solidifies (*9*, *17*–*19*). The transition from orderly to disorderly motion from the DM to PZ is necessary for proper body elongation (*1*, *9*, *20*). Excessively disordered motion in the DM [via experimental inhibition of bone morphogenetic protein (BMP) or fibroblast growth factor (FGF) signaling] impairs the flow of cells through the tailbud leading to a short body axis. Excessively ordered motion in the PZ (induced by moderate Wnt inhibition) produces prolonged anisotropic fluxes, unequal allotment of cells to the left or right PSM, and a bent body axis. Thus, understanding robustness and reproducibility of vertebrate body elongation requires understanding the nature of these tailbud cell state transitions.

In this study, we first define the trajectory of cell states in the zebrafish tailbud during body elongation using dimensional reduction algorithms and then segment the trajectory using a change point detection algorithm. We define gene expression state using single-cell RNA sequencing (scRNA-seq). We validate this algorithmic definition of cell states by comparing wild type and embryos with reduced Wnt, FGF, and BMP signaling and verify quantitative differences in gene expression cell states by multicolor fluorescent in situ hybridization. Next, we identify cell motion states by analyzing cell tracking data using a similar approach. We find that the cell tracking data can be segmented into reproducible cell motion states. We then perform an analysis of the pattern of cell motion states, as these datasets allow direct quantification of cell state dynamics over time. The pattern of cell motion state transitions in a 2- to 3-hour time average are the same in each wild-type embryo, indicating the reproducibility of the cell state dynamics. In addition, the cell state pattern for a single time point is typically the same as the pattern of a 2- to 3-hour time average, revealing some dynamic stability of this pattern. However, individual embryos exhibit transient deviations from the average pattern of cell states. Analysis of these transient deviations reveals them to be due to irregular collective cell migration, which produces bilaterally asymmetric convergence to the midline in the PSM. Thus, both the reproducible pattern of cell motion states and bilateral symmetry arise from temporal averaging. More generally, these results indicate that collective cell behavior does not necessarily buffer noisy individual cell behavior and that collective cell behavior can be a source of inappropriate asymmetry. Prospectively, our approach using a dimensional reduction method and a change point detection algorithm may prove useful in the analysis of other complex time series datasets.

## RESULTS

### Gene expression states

We performed scRNA-seq on dissected tails from 10 to 12 somite stage zebrafish embryos (Fig. 1A). We used wild-type embryos and embryos subject to treatments known to alter tailbud cell migration, specifically inhibition of FGF, BMP, or Wnt signaling (*1*, *9*, *13*, *20*). For each treatment, we prepared four biological replicates, each consisting of 10 to 12 tailbuds and resulting in 30,000 to 35,000 single-cell profiles. In a uniform manifold approximation and projection (UMAP) dimension reduction plot of wild type, the neuronal and paraxial mesoderm form one large cluster with more differentiated cells at each end and common progenitors (cyan) in the middle [Fig. 1B (arrow) and fig. S1]. Wild-type and experimental samples consist of the same cell transcription profiles highlighting the robustness of gene expression states (Fig. 1D). This result is also consistent with previous scRNA-seq analysis of zebrafish embryos, indicating that perturbation of cell signaling does not create novel cell transcription profiles (*21*, *22*).

To enable direct quantitative comparisons between experimental conditions, we pooled the data from all wild-type and experimental replicates and created one unified pseudotime to define a single standard for classifying cells. Specifically, the cells in the main cluster were aligned along a neuronal-mesodermal axis from *sox3* expressing neuronal cells to *mespaa* expressing anterior PSM cells (Fig. 1B, arrow) (*22*). This approach avoids the requirement to define the NMP population a priori. Instead, NMPs will be located in the middle of the pseudotime sequence, and differentiation will proceed toward both ends, i.e., neuronal to the left and mesodermal to the right (Fig. 1C). Marker genes for neuronal and mesodermal development map with respect to pseudotime in the correct developmental sequence, indicating that the procedure was successful.

To define gene expression states, we extracted the wild-type data and then used a change point detection algorithm to divide pseudotime into a series of distinct states (*23*). The change point algorithm identified five transition points (fig. S2). These transition points (vertical lines in Fig. 1C) divide the pseudotime sequence into six states that generally agree with those predicted previously from marker gene expression (*24*). The states include a neural state, NMPs, and a succession of mesodermal states. The transition points were then mapped to the full pseudotime sequence, and we calculated the relative abundance of each state in wild-type, Wnt-inhibited embryos, FGF-inhibited embryos, and BMP-inhibited embryos (Fig. 1D).

**Fig. 2.**
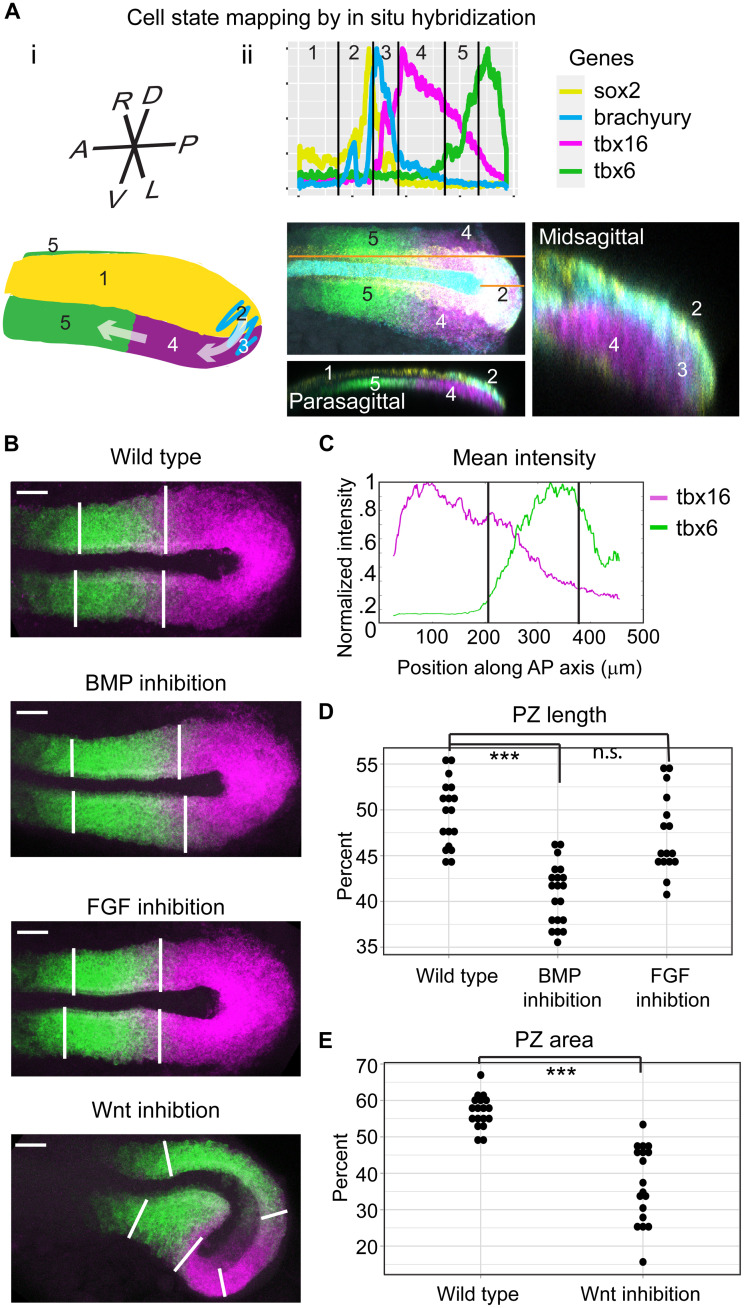
scRNA-seq gene expression states map to the tailbud. (**A**) (i) A schematic showing the developmental trajectory of the paraxial mesoderm in the tailbud. All panels show the expression of *sox2* (yellow), *brachyury* (cyan), *tbx16* (magenta), and *tbx6* (green). (ii) Fluorescent in situ hybridization maps the transcriptional states (numbered) defined by scRNA-seq onto the tailbud. In the dorsal view, the orange lines mark locations of the midsagittal (short line) and parasagittal (long line) slices. (**B**) Expression of *tbx16* (magenta) and *tbx6* (green). Vertical lines mark the transition from PZ to PSM and PSM to anterior PSM. Scale bars, 50 μm. (**C**) Plot of signal intensity in a representative wild-type embryo along the anterior-posterior axis. Vertical bars are cutoffs for the PZ and PSM of 20 and 85% of maximum *tbx6* expression, respectively. (**D**) PZ length normalized to total length of PZ and PSM in wild-type, BMP-inhibited, and FGF-inhibited embryos. (**E**) PZ area normalized to total area in wild-type and Wnt–inhibited embryos. ***P < 0.001. n.s., not significant. See also fig. S3.

To determine whether this analysis of scRNA-seq data accurately quantifies changes in cell state, we mapped the transcriptional states back onto the embryo and measured their abundance using multicolor fluorescent in situ hybridization for marker genes for the first five states (Fig. 2A). *Sox2* single–positive cells localize in the neural tube (state 1). *Sox2*- and *brachyury*-positive NMPs (state 2) occupy the DM. Nascent mesodermal progenitors (state 3) expressing *brachyury* and *tbx16* are located immediately ventral to the DM in the medial PZ. Mesodermal progenitors in the PZ (state 4) are *tbx16* single–positive cells located in the ventral and lateral tailbud. The PSM (state 5) is anterior to the transition from *tbx16* to *tbx6* expression.

To validate the scRNA-seq analysis, we chose to test the predictions of changes in the abundance of neuronal and PZ states. First, the scRNA-seq predicts that Wnt-inhibited embryos would have more neuronal cells (Fig. 1E). This is consistent with reports that elimination of Wnt signaling leads NMPs to exclusively adopt a neuronal fate (*3*). In our partial inhibition of Wnt signaling, 6 of 16 embryos have an abnormal cap of neuronal tissue covering the embryos’ posterior, confirming the scRNA-seq results (fig. S3).

A second prediction of the scRNA-seq analysis is that the PZ is smaller in BMP- and Wnt-inhibited embryos but not in embryos subject to FGF inhibition. To test this prediction, we performed fluorescent in situ hybridization for a PZ marker, *tbx16*, and a PSM marker, *tbx6* (Fig. 2B). In wild-type, BMP-inhibited, and FGF-inhibited embryos, the *tbx16* and *tbx6* signal was measured along the anterior-posterior axis of the embryo for both the left and right sides (Fig. 2C). The PZ/PSM transition was set to the value derived from the scRNA-seq analysis (20% of the maximum value of *tbx6*), and then, the PZ length was normalized to the total tail length (85% of maximum value of *tbx6*). Consistent with the scRNA-seq analysis, BMP- but not FGF-inhibited embryos exhibited a decrease in PZ length (Fig. 2D). Because of the bent body axis exhibited by most Wnt-inhibited embryos, the area of the PZ and PSM were quantified. As predicted, Wnt-inhibited embryos have a smaller PZ (Fig. 2E). Thus, this approach to analyzing scRNA-seq data accurately identifies cell states that can be quantitatively mapped back onto the embryo.

### Cell motion states

**Fig. 3.**
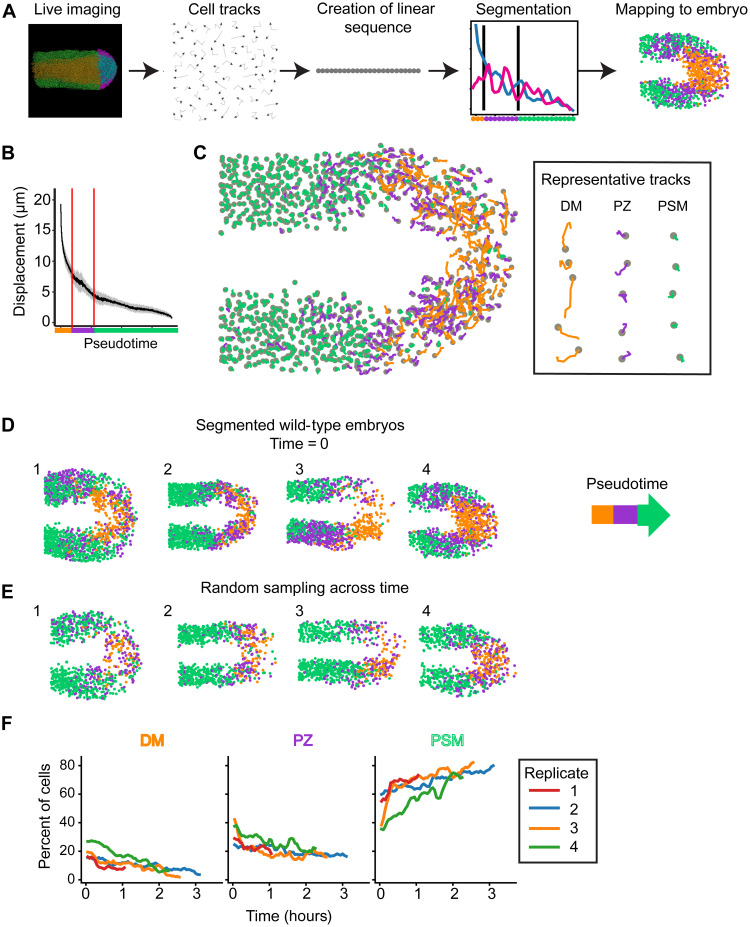
Cell motion states can be defined similarly to gene expression states. (**A**) Schematic of experimental approach. Embryos were imaged with a confocal microscope, and cells were tracked. Tracks were arranged into a one-dimensional pseudotime sequence and then segmented. Last, they were mapped back onto the embryo. (**B**) Plot of track displacement over pseudotime. The line is the sliding window mean of 100 tracks, and shading is the SD. Vertical lines mark transitions between states. (**C**) Tracks mapped back onto the embryo. Tracks are colored by pseudotime segmentation. Gray circles mark cell positions at the end of the interval. Representative tracks were chosen at random. (**D** and **E**) Four segmented wild-type replicates. Dots represent position of the cells at the start of the track. Colors represent pseudotime segment. Tracks are either chosen from the start of the time lapse (D) or randomly sampled from throughout the time lapse (E). (**F**) Abundance of cell motion states in each replicate at each time point.

We hypothesized that similar algorithms to those used to classify gene expression states could be applied to cell motion data to define the cell motion states (Fig. 3A). For this purpose, we used cell tracking data from confocal time-lapse imaging of cells in the DM through PSM collected over 1 to 3 hours in wild-type embryos, 7 to 10 somite embryos, and embryos subject to signaling perturbations (*9*, *13*, *20*). As with the gene expression analysis, a cell motion trajectory for each cell track was used to order the tracks in pseudotime, and the state transitions were defined using the change point detection algorithm and the cell motion statistics such as speed and displacement in a specific duration. The cell states were color coded and spatially mapped back onto the embryo using the original cell track position.

Because this is a novel approach, we separately optimized pseudotime assembly and segmentation starting with the wild-type embryos. Unlike gene expression, cell motion cannot be practically characterized instantaneously, and the choice of track interval involves trade-offs. Longer tracks individually contain more information, but the embryo contains fewer of them, and they may average out within-track transitions in cell motion state. We chose to use a sliding window of eight time points (21 min) as an empirically derived base unit. For each track, we calculated a distance matrix in which each element is equal to the cell’s displacement between the *i*-th and *j*-th time points. To form a linear sequence, we used a variational autoencoder (VAE), a neural network–based machine learning method, as a dimensional reduction tool. It embedded distance matrices from four wild-type embryos into a one-dimensional latent space. To construct a pseudotime sequence for each embryo, cells were assigned the rank of their latent space coordinate (*z*). This procedure successfully reproduced the known developmental sequence (fig. S4). It indicates that there is a continuity in cell migratory behavior in the tailbud, like that of gene expression. Cells that have similar cell motion traits are closer to each other in development.

To segment the pseudotime sequences, we sought parameters with a large variance over pseudotime. We found that either cell speed and track straightness (fig. S5) or, more simply, track displacement (Fig. 3, B and C) gives the best results. The change point detection algorithm classifies the tracks into three cell motion states (fig. S6). We mapped these states back onto the embryo by plotting cell position at the start of the track and taking either a single time point (Fig. 3D) or a time average created by randomly sampling tracks throughout the time lapse (Fig. 3E). The time averages are reproducible between embryos. Each embryo is segmented into a high-displacement state principally located in the DM, an intermediate displacement state mostly located in the PZ, and a low cell motion state mostly in the PSM (Fig. 3, D and E, and movie S1). These results are consistent with previous manual segmentations of the tailbud into DM, PZ, and PSM (*9*).

**Fig. 4.**
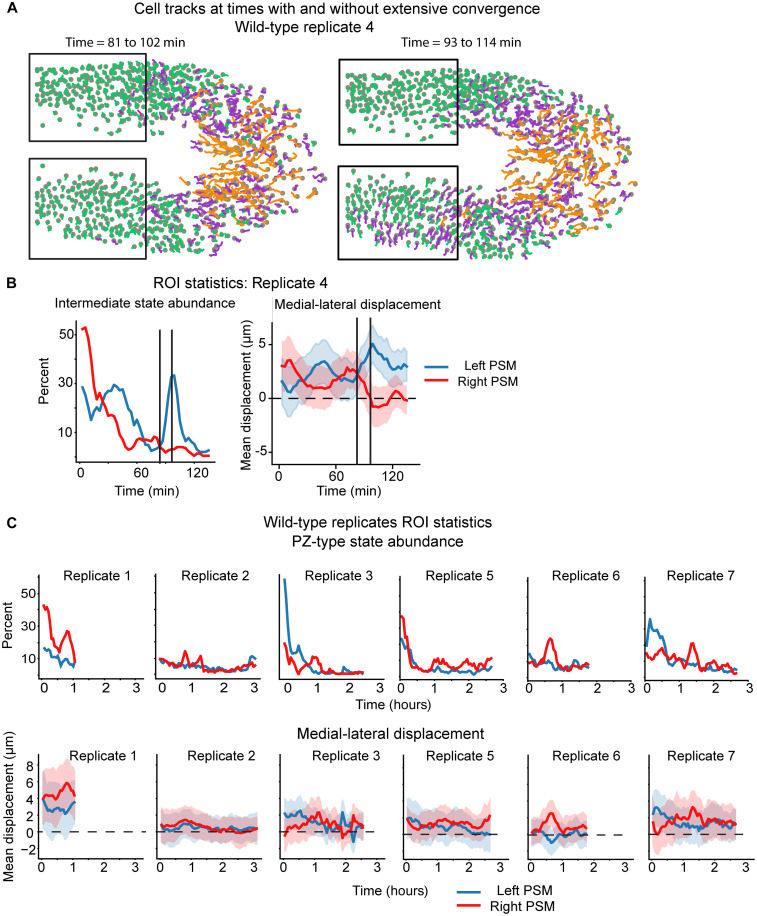
PSM exhibits significant left-right asymmetry. (**A**) Cell tracks in replicated four from two different times points plotted as in Fig. 3B. Boxes mark ROIs for left and right PSM region. (**B**) Track statistics for cells in left and right PSM ROIs in embryo shown in (A). The left plot is abundance of PZ-type tracks [magenta color in (A)] in the PSM over time. The right plot is track displacement along the medial-lateral axis (mean and SD) over time. Positive numbers are moving toward the midline. (**C**) PSM region ROI statistics for the other wild-type replicates. PSM convergent behavior differs markedly between embryos and between left and right sides within embryos.

We next analyzed the temporal dynamics of the cell motion states. We find that the relative abundance of PSM-type tracks increases over time, while the DM and PZ shrink (Fig. 3F). This is consistent with the gradual decrease in the size of the tailbud as progenitor cells (DM and PZ) differentiate into PSM faster than new progenitors are created (*8*, *25*). However, we observed notable fluctuations in cell state size in some embryos. For example, in wild-type embryo 4, patches of cells transition rapidly from PZ to PSM– and then to PSM to PZ–type movements (Fig. 4A and movie S1). These fluctuations are asymmetric between the left and right PSM and appear to be tied to differences in collective cell motion as they convergence toward the midline (Fig. 4A). To quantify this effect, we created regions of interest (ROIs) for the left and right anterior PSM and measured the abundance of PZ-type tracks and track displacement along the medial-lateral axis (Fig. 4, B and C). We found large differences between experimental replicates. Two embryos (replicates 3 and 4) have antiphase oscillations in medial-lateral displacement between the left and right PSM, where one side efficiently converges toward the midline, while the other has less convergence or even a net lateral motion. These fluctuations roughly correlate with differences in the abundance of PZ-type tracks. Replicate 1 exhibits constant convergence in both the left and right PSM, although the right side is moving significantly faster (*P* < 0.001) and has more PZ-type tracks. In contrast, replicate 2 has no net movement toward the midline in either PSM and a constant, low abundance of PZ-type tracks. Given this variability, we generated three more wild-type replicates (numbers 5 to 7). Their cells were assigned pseudotime coordinates by mapping them to the nearest tracks in the preestablished pseudotime sequence. They recapitulate the original phenotypes. Thus, cell motion state abundance is variable over time within a single embryo even while the average pattern in each embryo is the same.

In addition to the creation of individual pseudotime sequences for each embryo, we also performed a pooled analysis by mapping all cell tracks in wild-type replicates 1 to 4 to one pseudotime sequence and then segmenting that sequence. This approach is analogous to the unified pseudotime assembly used for the scRNA-seq data. Using this approach yields the same results as separate segmentations for three of four replicates (fig. S7). The outlier is replicate 1 where almost the entire PSM is classified as PZ-type tracks. This indicates that the converging cells of the PSM in this replicate move similarly to the high-displacement PZ cells in the other embryos. However, within a single replicate, the PZ and PSM do have different migratory statistics (fig. S7), indicating that the relative differences between cell motion states is reproducible from embryo to embryo, whereas the absolute magnitude of the differences may vary (*9*). Thus, individual versus pooled analyses provide different information, e.g., average cell state patterns, differences within a single embryo over time, and differences among embryos.

### The effect of signaling perturbations on cell motion state

**Fig. 5.**
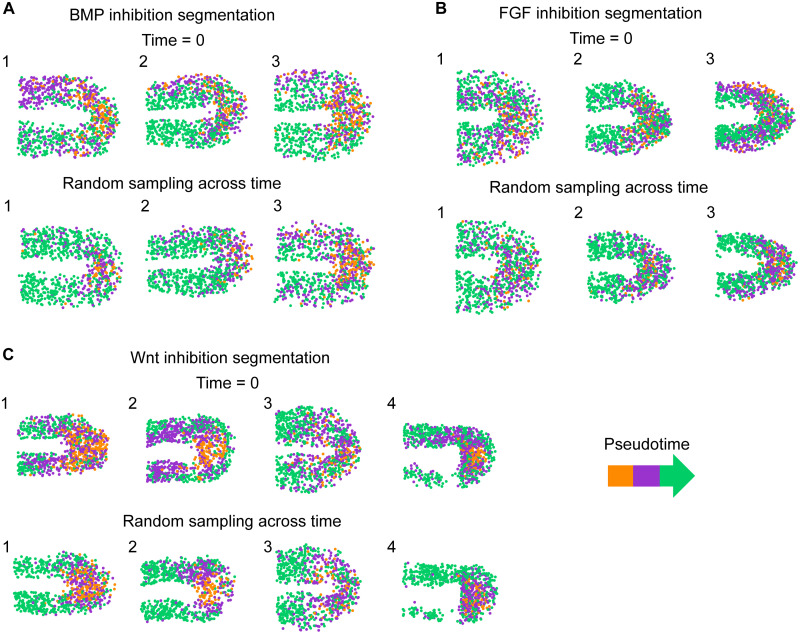
Cell motion segmentation in embryos subject to signaling perturbations. BMP-inhibited (**A**), FGF-inhibited (**B**), and Wnt-inhibited (**C**) embryo cell tracks were mapped into the pseudotime sequence and then segmented using the same algorithms as the wild-type embryos. Tracks were mapped onto the embryos. Dots represent position of the cells at the start of the track. Colors represent pseudotime segment. For plotting, tracks were chosen from either the first time point or randomly sampled from across the time lapse.

We next sought to determine whether signaling perturbations known to affect cell migratory behaviors would alter the cell state transitions. To this end, we took cell tracks from embryos subject to FGF, Wnt, or BMP signaling inhibition and assigned them pseudotime coordinates by mapping them to the most similar wild-type tracks. We then colored the cells by pseudotime and segmented them using the change point detection algorithm (fig. S4 and Fig. 5). The pseudotime sequence largely agrees with the developmental sequence, although FGF signaling– and BMP signaling–inhibited embryos have a noisier pattern of cell motion states than wild-type embryos (Fig. 5, A and B). This result is consistent with the observation that BMP inhibition suppresses the average differences in speed and track straightness between the DM, PZ, and PSM regions (*9*, *20*). However, the use of additional factors in the VAE algorithm likely aids in recovery of developmental sequences. Wnt signaling inhibition, a perturbation that affects the coordination of cell movement more than its cell-autonomous characteristics (*1*, *9*), largely retains the wild-type cell motion state pattern (Fig. 5C). These results indicate that similarly to scRNA-seq, the embryos’ cell motion statistics retain sufficient tissue identity to allow for data integration.

**Fig. 6.**
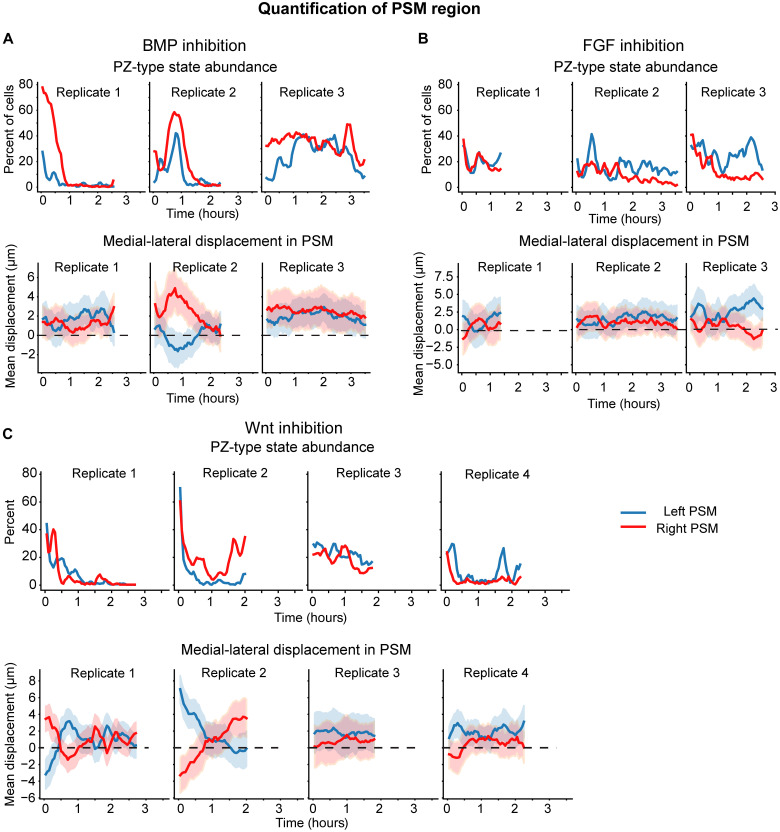
PSM region cell motion dynamics after BMP, FGF, or Wnt signaling inhibition. Track statistics in ROIs of the left and right PSM after (**A**) BMP inhibition, (**B**) FGF inhibition, or (**C**) Wnt inhibition. The first row in each panel displays the abundance of PZ-type (magenta colored) tracks in the PSM over time. Second row shows medial-lateral displacement (mean and SD) over time. Positive numbers represent movement toward the midline.

Inhibition of BMP or FGF signaling decreases coordination of cell motion in the DM, while inhibition of Wnt signaling prolongs left-right asymmetries in cell flux in the PZ leading to a bent body axis (*1*, *9*). We hypothesized that these perturbations could have similar effects in the PSM. Therefore, we repeated the measurements for PZ-type track abundance and medial-lateral displacement in the PSM in these backgrounds (Fig. 6). BMP- and FGF-inhibited embryos have fluctuations in the abundance of PZ-type tracks and mean medial-lateral displacement similar to wild type, suggesting that this process is not regulated by these signals (Fig. 6, A and B). In the Wnt signaling inhibition case, three of the four embryos exhibited wild-type patterns of antiphase oscillations or sustained but low magnitude differences in convergence. They also generally had similar numbers of PZ-type tracks in the left and right PSM. The outlier, replicate 2, had major asymmetries where the left side was initially moving medially, while the right side moved in a posterior-lateral direction. Subsequently, the left PSM cells ceased motion and adopted almost exclusively PSM-type tracks, while the right PSM began moving medially. This was accompanied by complementary curvatures of the PSM-notochord interfaces. Thus, while embryos subject to moderate Wnt signaling inhibition generally have wild-type PSM behaviors, they can exhibit abnormalities that correlate with gross morphological defects.

## DISCUSSION

The ability to perform automated classifications of cells into distinct states is a powerful tool both for organizing data and the discovery of underlying principles. Extensive effort has gone into developing techniques for identifying gene expression states from scRNA-seq data (*26*). Meanwhile, cell motion has traditionally been described more qualitatively. Our parallel analysis of gene expression and cell migration states using dimensional reduction algorithm followed by a change point detection algorithm demonstrates that these cell state transitions can be similarly systematically defined and mapped back onto the embryo.

Gene expression and cell motion states not only share some similarities but also have many differences. The gene expression transitions are spatially segregated and reproducible. They can be mapped onto the embryo using in situ hybridization (Figs. 1 and 2). The PZ-PSM transition is bilaterally symmetric and reproducible across 10 to 12 somite wild-type embryos (Fig. 2). Signaling perturbations do not create new states but do change the abundance of wild-type states. This phenomenon has been observed in other developmental contexts (*27*). Overall, gene expression states are very robust.

**Fig. 7.**
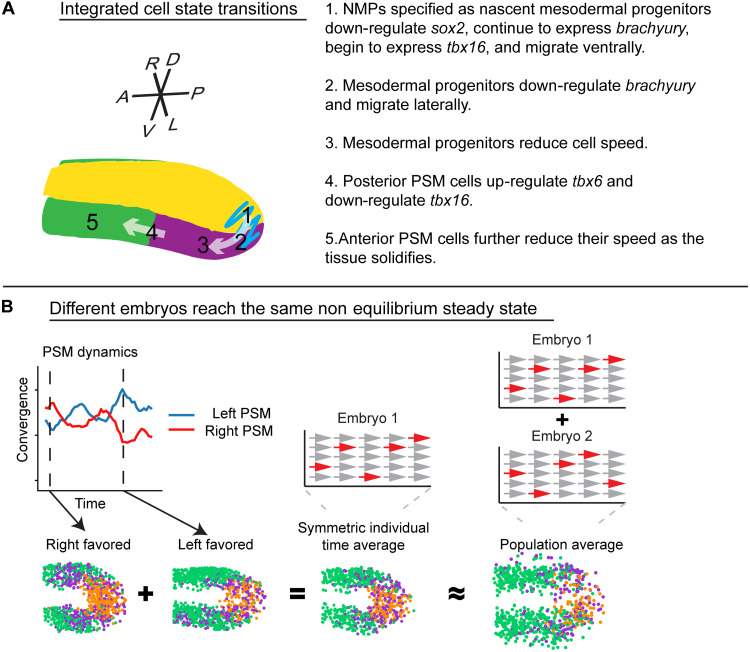
Summary of cell state transitions. (**A**) Illustration of gene expression states. (**B**) Schematic of how time averaging creates consistent, left-right symmetric development both in individual embryos and in populations.

The cell motion states have a somewhat different spatial pattern than the gene expression states. The transitions between DM, PZ, and PSM are roughly in the same position as the gene expression transitions but not always exactly (Fig. 7A). This is likely due to time delays inherent in mRNA processing and translation, posttranscriptional gene regulation, and physical constraints on cell motion. The pattern of cell motion transitions is also spatially noisier than for gene expression states. Some of this difference is technical. Cell motion has far fewer parameters than gene expression. However, much of the noise is biological and due to changes in collective cell behavior, not just the behavior of single cells. While our data do indicate a slowing of cell motion during PSM solidification from the anterior to the posterior, counter examples of the anterior PSM cells moving with a relatively rapid and processive PZ-type motion while the posterior PSM is more static can be readily identified. Thus, PSM solidification does not occur in a smooth anterior to posterior progression. These fluctuations between PZ and PSM type motion occur rapidly and involve small patches of tissue, suggesting that the anterior PSM remains close to the fluid-to-solid jamming transition (*19*).

Creation of a straight body axis requires equally sized left and right paraxial mesoderm. However, paradoxically, the two PSMs often have divergent behaviors in both cell speed and directionality. These sorts of asymmetries have also been observed in the nascent PSM during gastrulation, suggesting that this is a general feature of zebrafish convergent extension (*28*). These results conflict with models of body elongation (derived from chick) where coordinated convergence movements in the PSMs compress the notochord, forcing it to elongate into the tailbud, thereby displacing PZ cells (*29*). Furthermore, the notochord can elongate faster than the paraxial mesoderm, such as when loss of *tbx16* prevents PZ cells from entering the PSM (*30*). In these mutants, the elongating notochord buckles. Thus, in zebrafish, notochord elongation does not appear to be driven by convergence of the PSM.

One method for achieving robust development from noisy processes is temporal averaging. If a parameter is integrated over a time span significantly longer than the period of fluctuations, then individual replicates will converge toward the mean (Fig. 7B). For gene expression states, cells are able to maintain their state in the face of transcriptional bursting by using the longer stability of proteins (*31*). Two drivers of PSM elongation are addition of cells to the PSM from the PZ and convergence of PSM cells. In wild-type embryos, the PZ has left-right cell movement oscillations on the order of tens of minutes (*1*). Wnt inhibition causes permanent asymmetries for an entire 3-hour time lapse and a bent body axis indicating that the reversals are necessary to evenly disperse mesodermal progenitors to the left and right side. We find similar fluctuations of convergence patterns in the PSM with periods on the order of tens of minutes to up to an hour. These fluctuations are not simply noisy single-cell behavior but rather abrupt changes in the collective motion of 10 to 100 cells. Thus, while a straight spine is essential for the biomechanics of the adult, transient small imbalances are tolerable in the embryo. How cell dynamics are tuned and averaged to obtain robust left-right symmetry remains an open question.

## MATERIALS AND METHODS

### Data and code availability

The scRNA-seq data have been archived at National Center for Biotechnology Information Gene Expression Omnibus (NCBI GEO; accession no. GSE173894).

### Zebrafish methods

Tüpfel long fin zebrafish were raised according to standard protocols and experiments approved by the Institutional Animal Care and Use Committee. Experiments were performed before sex determination in zebrafish (*32*). FGF, BMP, and Wnt signaling perturbations were performed using protocols previously developed to modulate cell migration (*9*, *20*). Specifically, starting at the six-somite stage, embryos were incubated in 50 mM SU5402 or 40 mM DMH1 for 2 hours to inhibit FGF or BMP signaling, respectively. Wnt signaling was inhibited by injecting *notum-1* mRNA at a concentration of 150 ng/ml into embryos at the single-cell stage and then incubating them until the 10-somite stage. This treatment yields a phenotypic spectrum, and embryos with nascent body elongation defects were chosen for further experiments (*20*).

### Tailbud dissections and scRNA-seq

Embryos were incubated until the 10- to 12-somite stage and then dissected in ice-cold Hank’s balanced salt solution (HBSS). The tail was collected by cutting immediately posterior to the last formed somite. Groups of tails consisting of 10 tails for wild-type, FGF inhibition, or BMP inhibition or 12 tails for Wnt inhibition were pooled together. Cells were dissociated by incubation in papain solution (20 U/ml; Worthing Biochemical) for 15 min at 29°C with gentle agitation. Halfway through the incubation, the solution was triturated 10 times with a P200 pipette. Cells were spun down at 300*g* for 5 min and then resuspended in 40 μl of cold HBSS. Cell concentration and viability were checked with a hemocytometer, and the volume of the solution was adjusted if required.

#### Construction of 10x Genomics single-cell 3′ RNA-seq libraries (version 3) and sequencing with an Illumina HiSeq4000

##### GEM generation and barcoding

Single-cell suspension in RT Master Mix was loaded on the Single Cell A Chip and partition with a pool of about 750,000 barcoded gel beads to form nanoliter-scale gel beads in emulsions (GEMs). Each gel bead has primers containing (i) an Illumina R1 sequence (read 1 sequencing primer), (ii) a 16-nt 10x barcode, (iii) a 10-nt unique molecular identifier (UMI), and (iv) a poly-dT primer sequence. Upon dissolution of the gel beads in a GEM, the primers are released and mixed with cell lysate and master mix. Incubation of the GEMs then produces barcoded, full-length cDNA from polyadenylated mRNA.

##### Post GEM-RT cleanup, cDNA amplification, and library construction

Silane magnetic beads were used to remove leftover biochemical reagents and primers from the post-GEM reaction mixture. Full-length, barcoded cDNA was then amplified by polymerase chain reaction (PCR) to generate sufficient mass for library construction. Enzymatic fragmentation and size selection were used to optimize the cDNA amplicon size before library construction. R1 was added to the molecules during GEM incubation. P5, P7, a sample index, and R2 (read 2 primer sequence) were added during library construction via end repair, A tailing, adaptor ligation, and PCR. The final libraries contain the P5 and P7 primers used in Illumina bridge amplification.

##### Sequencing libraries

The single-cell 3′ protocol produces Illumina-ready sequencing libraries. A single-cell 3′ library comprises standard Illumina paired-end constructs, which begin and end with P5 and P7. The single cell 3′ 16–base pair (bp) 10x barcode and 10-bp UMI are encoded in R1, while R2 is used to sequence the cDNA fragment. Sequencing a single-cell 3′ library produces a standard Illumina BCL data output folder. The BCL data include the paired-end R1 (containing the 16-bp 10x barcode and 10-bp UMI) and R2 and the sample index in the i7 index read.

### Preprocessing of scRNA-seq data

We aligned the scRNA-seq data to Grcz11 and demultiplexed using Cell Ranger (10x Genomics). After the generation of expression matrices for each sample, we used Seurat v3 for preprocessing and clustering of scRNA-seq data (*27*). First, we excluded cells with an ectopic number of genes or exceeding a specified percentage of mitochondrial genes based on visual inspection for the distribution of these statistics. After the filtering genes, we conducted integration following Seurat’s SCTransform integration (*33*).

We applied principal components analysis (PCA) and embedded the 30-dimensional PCA coordinates into two-dimensional UMAP. We clustered cells by Seurat function “FindClusters” with a resolution parameter of 0.5.

### Pseudotime estimation of scRNA-seq

To recover cell state dynamics encoded in the gene expression data, we ordered a subset of scRNA-seq cells that belong to the axis from neural tube to PSM so that its ordering recapitulates the developmental trajectory during body elongation. In particular, we embedded the *z* scores of 30-dimensional PCA coordinates of cells belonging to specified clusters (*Sox3*^+^, *Sox2*^+^, DM, PZ, pPSM, and aPSM) into one-dimensional UMAP coordinates. Here, we expected that the most variable axis within gene expression space during this process would be the developmental trajectory. For UMAP embedding, we used the “umap-learn” package in Python and set “n_neighbors” as 400 and “min_dist” as 0.1.

### Segmentation of scRNA-seq pseudotime

We segmented the pseudotime trajectory of scRNA-seq into several segments within which each cell *c* has similar *z* scores of 30-dimentional PCA coordinate *x_c_* to dissect the dynamics along the progression of cell state transitions during zebrafish body elongation. We used a Bayesian algorithm of change detection (*23*) to find break points of segments *b_k_*(*k* = 1, …, *K*), which minimize the total error from the mean of profile of the segment ∑*_k_E_k_* where $E_{k}=\sum_{c\in C_{k}}V_{c},V_{c}={||x_{c}-\mu_{k}||}^{2},\mu_{k}=\frac{1}{|C_{k}|}\sum_{c\in C_{k}}x_{c},C_{k}=\{c|b_{k-1}<\tau_{c}<b_{k}\}$, and τ*_c_* is the discretized rank of the estimated pseudotime. We discretized the pseudotime rank into 30 bins for computational efficiency. We determined *K* as five scRNA-seq data using the elbow method (*34*), which chose a saturation point along the group variation curve as a function of the number of clusters.

### Estimation and segmentation of cell movement pseudotime

To recover cell state changes along the developmental axis for the cell motion, we also ordered cell track trajectory into one-dimensional pseudotime. It was constructed using four wild-type embryos. For this purpose, we applied a VAE (*35*) to distance matrices of cell trajectories between eight subsequent time points. We defined the input for cell *c* at time point *t* as the distance matrix *D*_*c*,*t*_ ∈ *R*^8×8^ between the cell *c*’s position at time point *t*, …, *t* + 7. Here, we assume that these distance matrices are generated from one-dimensional latent cell state *z* ∈ *R*

$$P(z_{c,t})=N(z_{c,t}|0,1)$$

$$P(D_{c,t}|z_{c,t})=N[D_{c,t}|\mu_{\theta }(z_{c,t}),\sigma_{\theta }^{2}(z_{c,t})]$$

where $\mu_{\theta },\sigma_{\theta }^{2}:R\to R^{8\times 8}$ is a decoder neural network with convolutional layers, the implementation details of which are shown in fig. S8.

To optimize this probabilistic model efficiently and approximate the posterior distribution *P*(*z*_*c*,*t*_ ∣ *D*_*c*, *t*_), we defined variational posterior distribution, $q_{\phi }(z_{c,t}|D_{c,t})=N[z_{c,t}|\mu_{\phi }(D_{c,t}),\sigma_{\phi }^{2}(D_{c,t})]$, where μ_ϕ_, $\sigma_{\phi }^{2}:R^{8\times 8}\to R$ are encoder neural network with convolutional layers, the implementation details of which are shown in fig. S8. Here, we derived evidence lower bound

$$L(\theta ,\phi )=E_{q_{\phi }(z_{c,t}|D_{c,t})}[\log P_{\theta }(D_{c,t}|z_{c,t})]+D_{KL}[q_{\phi }(z_{c,t}|D_{c,t})|P_{\theta }(z_{c,t})]$$

$$\approx \log P_{\theta }(D_{c,t}|{\tilde{z}}_{\phi ,c,t})+D_{KL}[q_{\phi }(z_{c,t}|D_{c,t})|P_{\theta }(z_{c,t})]$$

where ${\tilde{z}}_{\phi ,c,t}$ are derived from *q*_ϕ_(*z*_*c*,*t*_ ∣ *D*_*c*,*t*_), using reparametrized sampling (*35*). We optimized parameters included in encoder and decoder networks using Adam implemented in PyTorch. We adopted early stopping with patience = 30, which was implemented in PyTorch lightning. We assigned *z*_*c*,*t*_ as a cell movement pseudotime of cell *c* at time point *t*. We segmented the cell movement pseudotime using the same methodology for the segmentation of scRNA-seq pseudotime, except that the properties *x*_*c*,*t*_ for minimizing within-group variation are the *z* scores of the track displacement or speed and track straightness for the eight time points. We specified the number of change points *K* as using the elbow method (*34*), which chose a saturation point along the group variation curve as a function of the number of clusters.

For three additional wild-type embryos and all embryos subject to signaling perturbations, we mapped their tracks into the preestablished pseudotime by assigning each track the pseudotime coordinate of the closest track in the pseudotime sequence. This approach produced consistent results and is more computationally efficient than continually reconstructing pseudotime each time new data are analyzed.

### PSM quantification

ROIs consisting of the anterior 125 μm of the left and right PSM were constructed. Tracks were assigned to the ROI if the cell was within the region at the start of the track. Mean displacement along the medial-lateral axis and the relative abundance of PZ-type tracks were calculated at each time point.

### Multicolor fluorescent in situ hybridization

Probes for *sox2*, *brachyury*, *tbx16*, and *tbx6* were purchased from Molecular Instruments. The hairpins and colors are listed in Table 1. Staining of 10 to 12 somite embryos was performed using their recommended protocol (*36*) with a few modifications. Specifically, batches of 15 embryos were stained simultaneously. The *tbx6* probe was diluted 1:10 to avoid excessive bleed through the *sox2* channel. DAPI (4′,6-diamidino-2-phenylindole) was added to the amplification mixture. After staining embryos were taken through a series of 25%/50%/75% glycerol in phosphate-buffered saline. The posterior half of the embryo was isolated and mounted dorsal side up in 75% glycerol. Embryos were imaged with a Zeiss LSM 880 Airyscan confocal microscope using a 20× objective.

**Table 1.** Fluorescent in situ hybridization reagents.

Gene	Hairpin	Dye
Sox2	B1	Alexa Fluor 546
Tbx16	B2	Alexa Fluor 647
Brachyury	B3	Alexa Fluor 488
Tbx6	B4	Alexa Fluor 594

Preprocessing of the microscopy images was done using ImageJ. The *sox2* and *tbx6* channels were subtracted from each other to eliminate bleed-through. Images were rotated to a consistent orientation, and a maximum intensity projection was created. Adaxial cells were identified in the DAPI channel and manually removed from the image. The midline separating the embryo into left and right halves was identified manually. Subsequent quantification was performed in MATLAB. The image was smoothed with a Gaussian filter, and the ROI was thresholded using Otsu’s algorithm on both the *tbx16* and *tbx6* channels. For wild-type, BMP-inhibited, and FGF-inhibited embryos, average fluorescent intensity was measured along the *x* axis of the image and normalized to the maximum value. This was done separately for the left and right sides of the embryo. The PZ/PSM boundary was taken to be the first point with a value greater than 20% of the maximum *tbx6* value. The anterior end of the PSM was defined as the last point greater than 85% of the maximum *tbx6* value. The scaled PZ length was the PZ length divided by the distance from the end of the tail to the anterior boundary of the PSM.

Wnt-inhibited embryos had some modifications to the quantification. In bent embryos, the boundary separating the left and right halves was taken to be a line through the midpoint of the tailbud *brachyury* signal to the notochord and then following the notochord toward the head. For wild-type and Wnt-inhibited embryos, the outer perimeter of the embryo was traced manually. The curve was smoothed with a Savitzky-Golay filter and defined as the embryo’s axis. Pixels in the ROI were mapped to their closest points on the perimeter using the distance2curve function from J. D’Errico. The mean intensity along the axis was calculated using a sliding window. The same thresholds were used for the PZ and PSM, as described previously. The boundaries for these regions were taken to be a perpendicular dropped from the axis at the cutoff point. The scaled PZ area was the PZ area divided by the area of the PZ plus PSM. Statistics were calculated using Mann-Whitney *U* test.

## Notes

View/request a protocol for this paper from *Bio-protocol*.

## References

[1] D. Das, V. Chatti, T. Emonet, S. A. Holley, Patterned disordered cell motion ensures vertebral column symmetry. Dev. Cell 42, 170–180.e5 (2017). 28743003 10.1016/j.devcel.2017.06.020 PMC5568629

[2] C. S. Simon, A.-K. Hadjantonakis, C. Schröter, Making lineage decisions with biological noise: Lessons from the early mouse embryo. Wiley Interdiscip. Rev. Dev. Biol. 7, e319 (2018). 29709110 10.1002/wdev.319 PMC6002940

[3] B. L. Martin, D. Kimelman, Canonical Wnt signaling dynamically controls multiple stem cell fate decisions during vertebrate body formation. Dev. Cell 22, 223–232 (2012). 22264734 10.1016/j.devcel.2011.11.001 PMC3465166

[4] E. Tzouanacou, A. Wegener, F. J. Wymeersch, V. Wilson, J. F. Nicolas, Redefining the progression of lineage segregations during mammalian embryogenesis by clonal analysis. Dev. Cell 17, 365–376 (2009). 19758561 10.1016/j.devcel.2009.08.002

[5] D. Jülich, R. Geisler, S. A. Holley, Integrinalpha5 and delta/notch signaling have complementary spatiotemporal requirements during zebrafish somitogenesis. Dev. Cell 8, 575–586 (2005). 15809039 10.1016/j.devcel.2005.01.016

[6] A. Agathon, C. Thisse, B. Thisse, The molecular nature of the zebrafish tail organizer. Nature 424, 448–452 (2003). 12879074 10.1038/nature01822

[7] M. Müller, E. v. Weizsäcker, J. A. Campos-Ortega, Expression domains of a zebrafish homologue of the Drosophila pair-rule gene hairy correspond to primordia of alternating somites. Development 122, 2071–2078 (1996). 8681788 10.1242/dev.122.7.2071

[8] A. Attardi, T. Fulton, M. Florescu, G. Shah, L. Muresan, M. O. Lenz, C. Lancaster, J. Huisken, A. van Oudenaarden, B. Steventon, Neuromesodermal progenitors are a conserved source of spinal cord with divergent growth dynamics. Development 145, dev166728 (2018). 30333213 10.1242/dev.166728 PMC6240315

[9] A. K. Lawton, A. Nandi, M. J. Stulberg, N. Dray, M. W. Sneddon, W. Pontius, T. Emonet, S. A. Holley, Regulated tissue fluidity steers zebrafish body elongation. Development 140, 573–582 (2013). 23293289 10.1242/dev.090381 PMC3561786

[10] B. Benazeraf, P. Francois, R. E. Baker, N. Denans, C. D. Little, O. Pourquié, A random cell motility gradient downstream of FGF controls elongation of an amniote embryo. Nature 466, 248–252 (2010). 20613841 10.1038/nature09151 PMC3118990

[11] J. P. Kanki, R. K. Ho, The development of the posterior body in zebrafish. Development 124, 881–893 (1997). 9043069 10.1242/dev.124.4.881

[12] B. Steventon, F. Duarte, R. Lagadec, S. Mazan, J.-F. Nicolas, E. Hirsinger, Species-specific contribution of volumetric growth and tissue convergence to posterior body elongation in vertebrates. Development 143, 1732–1741 (2016). 26989170 10.1242/dev.126375

[13] N. Dray, A. Lawton, A. Nandi, D. Jülich, T. Emonet, S. A. Holley, Cell-fibronectin interactions propel vertebrate trunk elongation via tissue mechanics. Curr. Biol. 23, 1335–1341 (2013). 23810535 10.1016/j.cub.2013.05.052 PMC3725194

[14] R. Fior, A. A. Maxwell, T. P. Ma, A. Vezzaro, C. B. Moens, S. L. Amacher, J. Lewis, L. Saúde, The differentiation and movement of presomitic mesoderm progenitor cells are controlled by Mesogenin 1. Development 139, 4656–4665 (2012). 23172917 10.1242/dev.078923 PMC3509727

[15] A. J. Manning, D. Kimelman, Tbx16 and Msgn1 are required to establish directional cell migration of zebrafish mesodermal progenitors. Dev. Biol. 406, 172–185 (2015). 26368502 10.1016/j.ydbio.2015.09.001 PMC4639448

[16] T. Yabe, S. Takada, Mesogenin causes embryonic mesoderm progenitors to differentiate during development of zebrafish tail somites. Dev. Biol. 370, 213–222 (2012). 22890044 10.1016/j.ydbio.2012.07.029

[17] P. McMillen, S. A. Holley, The tissue mechanics of vertebrate body elongation and segmentation. Curr. Opin. Genet. Dev. 32, 106–111 (2015). 25796079 10.1016/j.gde.2015.02.005 PMC4470730

[18] L. Thomson, L. Muresan, B. Steventon, The zebrafish presomitic mesoderm elongates through compaction-extension. Cells Dev. 168, 203748 (2021). 34597846 10.1016/j.cdev.2021.203748 PMC7612712

[19] A. Mongera, P. Rowghanian, H. J. Gustafson, E. Shelton, D. A. Kealhofer, E. K. Carn, F. Serwane, A. A. Lucio, J. Giammona, O. Campàs, A fluid-to-solid jamming transition underlies vertebrate body axis elongation. Nature 561, 401–405 (2018). 30185907 10.1038/s41586-018-0479-2 PMC6148385

[20] D. Das, D. Jülich, J. Schwendinger-Schreck, E. Guillon, A. K. Lawton, N. Dray, T. Emonet, C. S. O’Hern, M. D. Shattuck, S. A. Holley, Organization of embryonic morphogenesis via mechanical information. Dev. Cell 49, 829–839.e5 (2019). 31178400 10.1016/j.devcel.2019.05.014 PMC6590525

[21] D. E. Wagner, C. Weinreb, Z. M. Collins, J. A. Briggs, S. G. Megason, A. M. Klein, Single-cell mapping of gene expression landscapes and lineage in the zebrafish embryo. Science 360, 981–987 (2018). 29700229 10.1126/science.aar4362 PMC6083445

[22] J. A. Farrell, Y. Wang, S. J. Riesenfeld, K. Shekhar, A. Regev, A. F. Schier, Single-cell reconstruction of developmental trajectories during zebrafish embryogenesis. Science 360, eaar3131 (2018). 29700225 10.1126/science.aar3131 PMC6247916

[23] P. Fearnhead, Exact and efficient Bayesian inference for multiple changepoint problems. Stat. Comput. 16, 203–213 (2006).

[24] M. Gouti, J. Delile, D. Stamataki, F. J. Wymeersch, Y. Huang, J. Kleinjung, V. Wilson, J. Briscoe, A gene regulatory network balances neural and mesoderm specification during vertebrate trunk development. Dev. Cell 41, 243–261.e7 (2017). 28457792 10.1016/j.devcel.2017.04.002 PMC5425255

[25] C. Gomez, E. M. Özbudak, J. Wunderlich, D. Baumann, J. Lewis, O. Pourquié, Control of segment number in vertebrate embryos. Nature 454, 335–339 (2008). 18563087 10.1038/nature07020

[26] B. Hie, J. Peters, S. K. Nyquist, A. K. Shalek, B. Berger, B. D. Bryson, Computational methods for single-cell RNA sequencing. Annu. Rev. Biomed. Data Sci. 3, 339–364 (2020).

[27] R. Satija, J. A. Farrell, D. Gennert, A. F. Schier, A. Regev, Spatial reconstruction of single-cell gene expression data. Nat. Biotechnol. 33, 495–502 (2015). 25867923 10.1038/nbt.3192 PMC4430369

[28] C. Yin, M. Kiskowski, P.-A. Pouille, E. Farge, L. Solnica-Krezel, Cooperation of polarized cell intercalations drives convergence and extension of presomitic mesoderm during zebrafish gastrulation. J. Cell Biol. 180, 221–232 (2008). 18195109 10.1083/jcb.200704150 PMC2213609

[29] F. Xiong, W. Ma, B. Bénazéraf, L. Mahadevan, O. Pourquié, Mechanical coupling coordinates the co-elongation of axial and paraxial tissues in avian embryos. Dev. Cell 55, 354–366.e5 (2020). 32918876 10.1016/j.devcel.2020.08.007 PMC7685225

[30] S. L. Amacher, C. B. Kimmel, Promoting notochord fate and repressing muscle development in zebrafish axial mesoderm. Development 125, 1397–1406 (1998). 9502721 10.1242/dev.125.8.1397

[31] A. Raj, C. S. Peskin, D. Tranchina, D. Y. Vargas, S. Tyagi, Stochastic mRNA synthesis in mammalian cells. PLOS Biol. 4, e309 (2006). 17048983 10.1371/journal.pbio.0040309 PMC1563489

[32] C. A. Wilson, S. K. High, B. M. McCluskey, A. Amores, Y.-l. Yan, T. A. Titus, J. L. Anderson, P. Batzel, M. J. Carvan 3rd, M. Schartl, J. H. Postlethwait, Wild sex in zebrafish: Loss of the natural sex determinant in domesticated strains. Genetics 198, 1291–1308 (2014). 25233988 10.1534/genetics.114.169284 PMC4224167

[33] C. Hafemeister, R. Satija, Normalization and variance stabilization of single-cell RNA-seq data using regularized negative binomial regression. Genome Biol. 20, 296 (2019). 31870423 10.1186/s13059-019-1874-1 PMC6927181

[34] T. S. Madhulatha, An overview on clustering methods. IOSR Journal of Engineering 2, 719–725 (2012).

[35] D. P. Kingma, M. Welling, Auto-encoding variational bayes. arXiv:1312.6114 [stat.ML] (2013).

[36] H. M. T. Choi, M. Schwarzkopf, M. E. Fornace, A. Acharya, G. Artavanis, J. Stegmaier, A. Cunha, N. A. Pierce, Third-generation in situ hybridization chain reaction: Multiplexed, quantitative, sensitive, versatile, robust. Development 145, dev165753 (2018). 29945988 10.1242/dev.165753 PMC6031405

