# Supplementary material for: Automated time-lapse data segmentation reveals in vivo cell state dynamics
Source: Sci Adv. 2023 Jun 2;9(22):eadf1814. doi: 10.1126/sciadv.adf1814 (PMC10413672; doi:10.1126/sciadv.adf1814)
Supplement: Supplementary file 1 — Figs. S1 to S8 Legend for movie S1 [file sciadv.adf1814_sm.pdf]

Science Advances
AAAS

Supplementary Materials for

Automated time-lapse data segmentation reveals in vivo cell state dynamics

Miriam A. Genuth *et al.*

Corresponding author: Scott A. Holley, scott.holley@yale.edu

Sci. Adv. **9**, eadf1814 (2023)
DOI: 10.1126/sciadv.adf1814

The PDF file includes:

Figs. S1 to S8
Legend for movie S1

Other Supplementary Material for this manuscript includes the following:

Movie S1

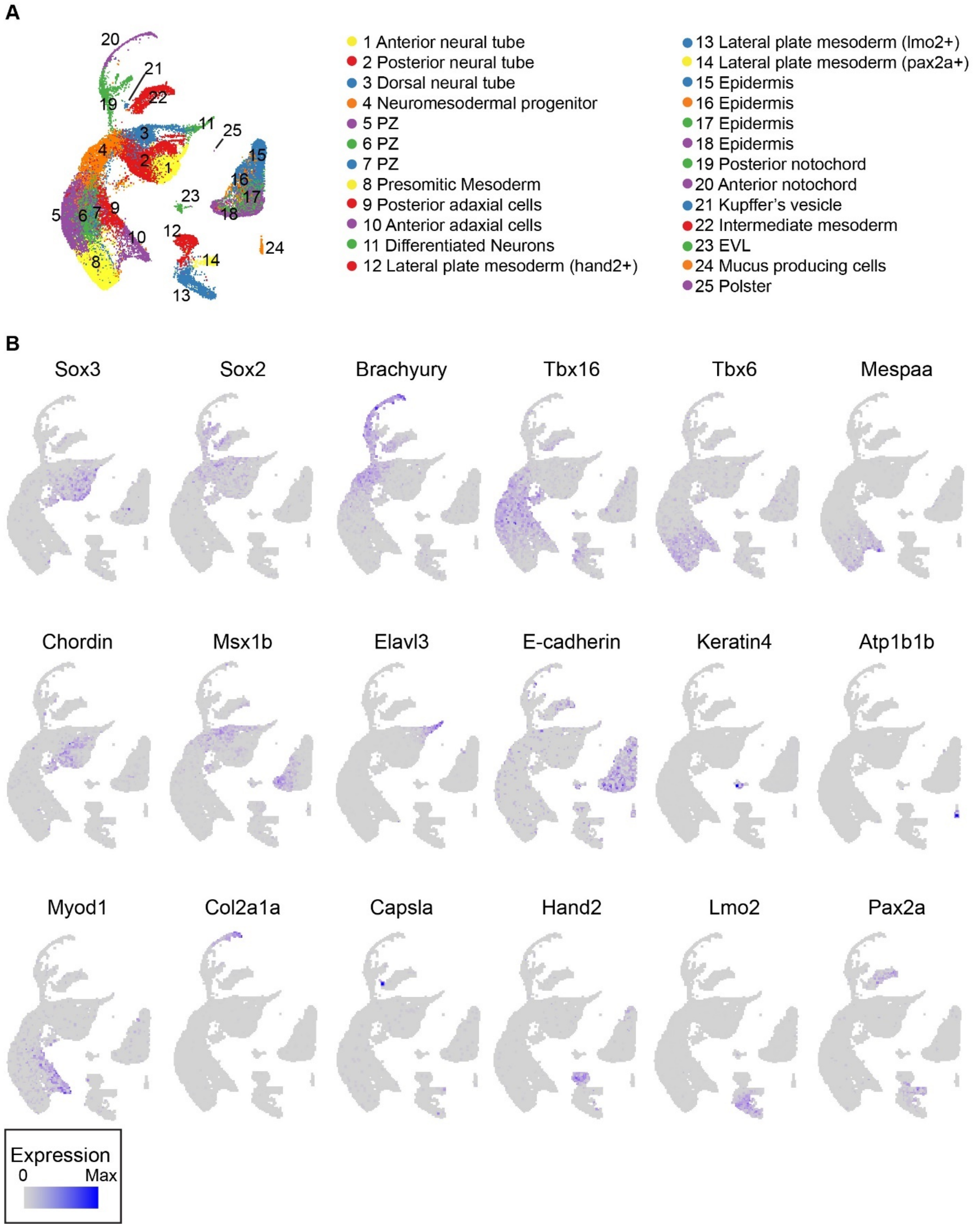

Fig S1. Gene expression states from scRNAseq.

(**A**) UMAP projection of cell clusters defined by Louvain clustering using Seurat. Clusters were manually annotated using marker genes. (**B**) UMAP projection of selected marker genes used to identify cell types.

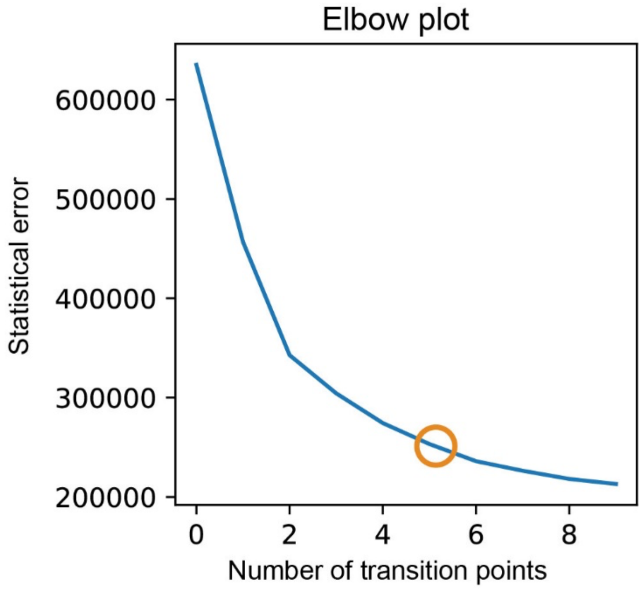

Fig S2. Gene expression pseudotime segmentation details.

Elbow plot used to select the number of transition points. Circle marks chosen value.

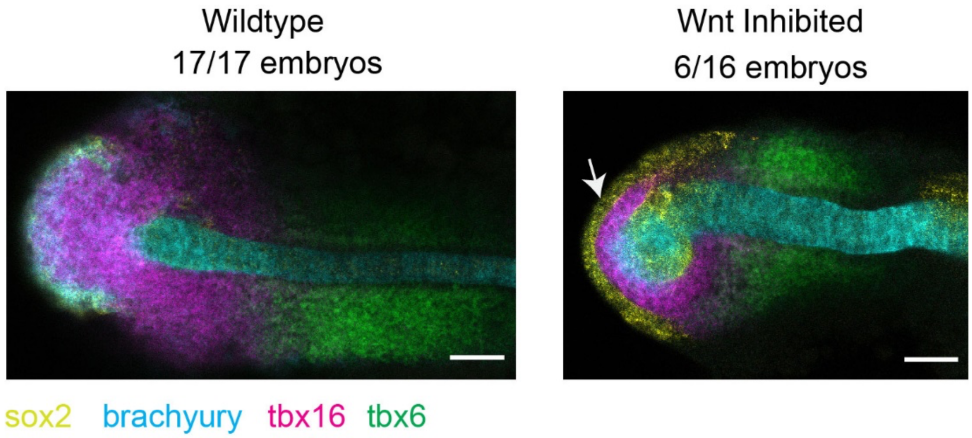

Fig S3. Wnt inhibited embryos have excessive neuronal tissue.

Five-micron projection through the ventral tailbud of a fluorescent in situ hybridization. Arrow points to inappropriately located sox2 single positive neuronal tissue. Scale bar= 50 microns

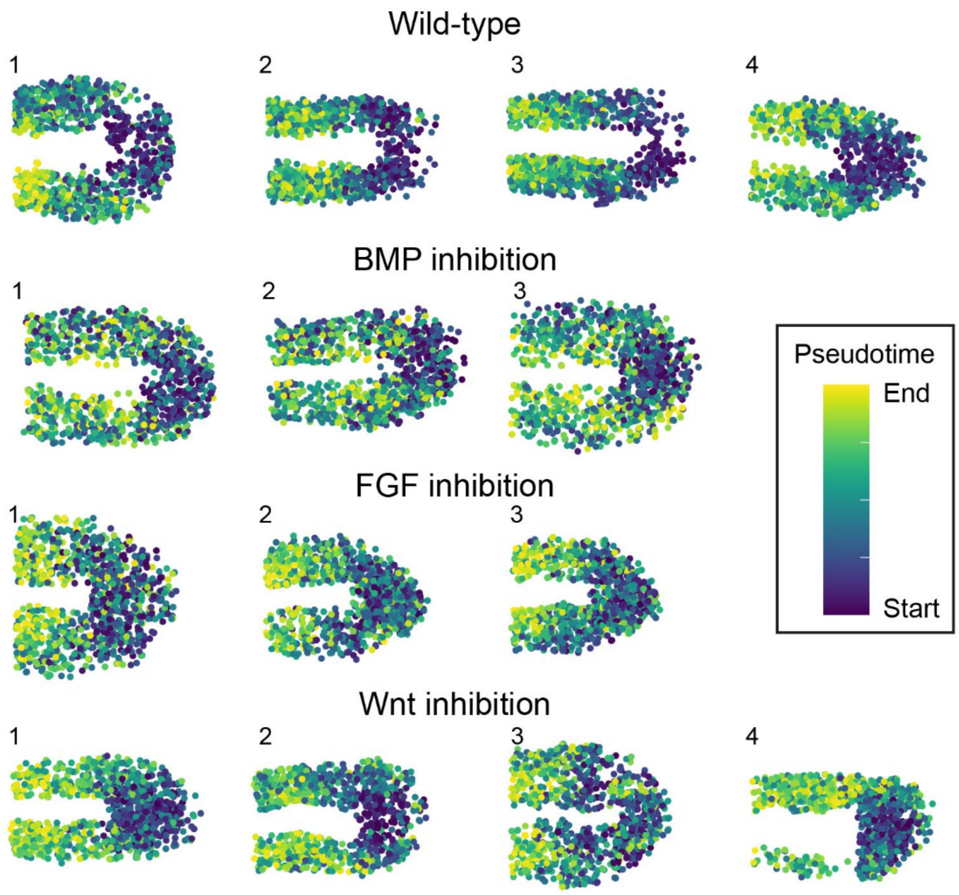

Fig S4. **Pseudotime generally reproduces the known developmental sequence.**

Cells randomly chosen from throughout the timelapse, a spot for each cell is created using the cell's position in x,y,z, and the spots are colored by pseudotime position.

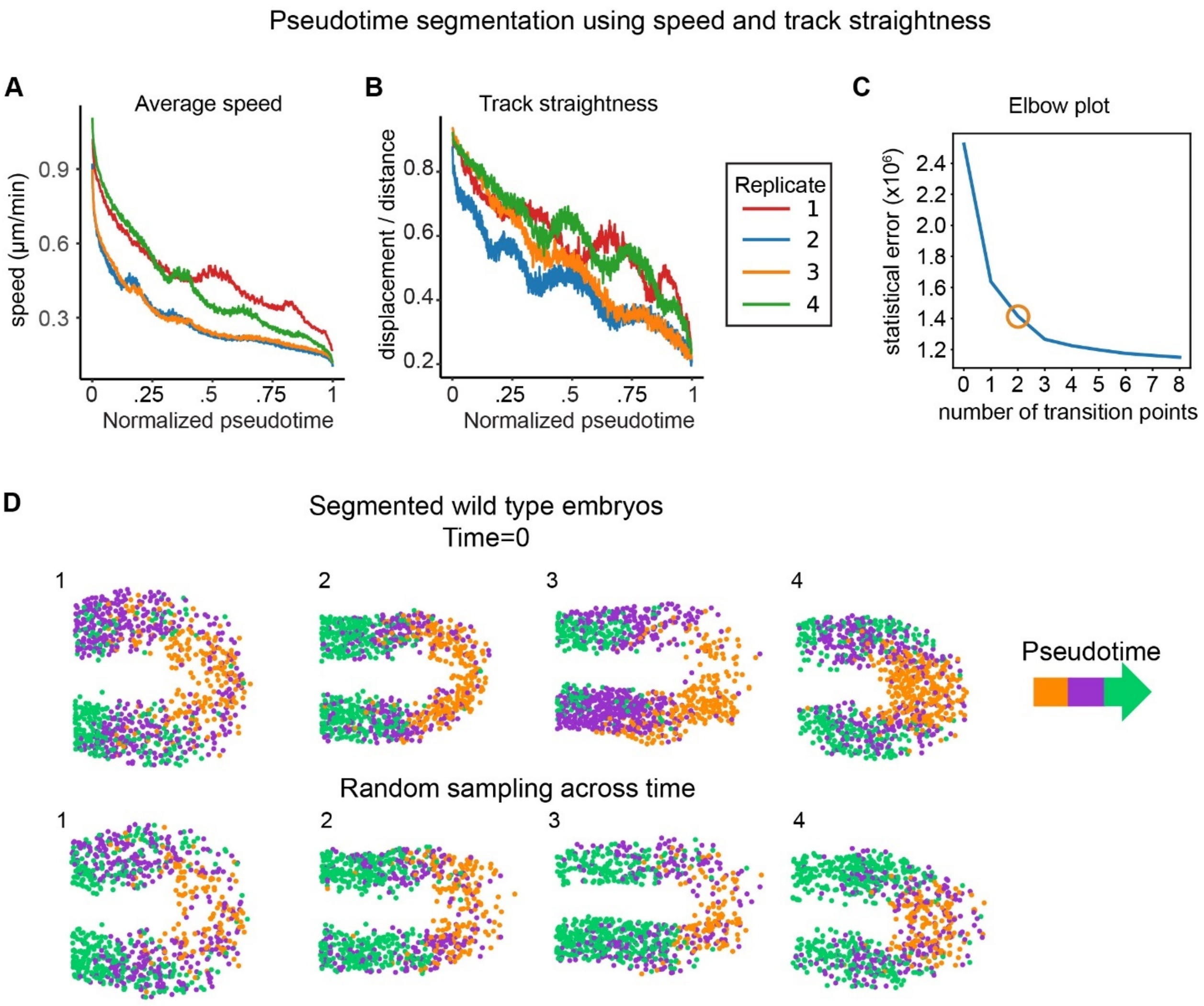

Fig S5. **Segmentation of pseudotime using speed and track straightness matches displacement** segmentation. (**A** and **B**) Track speed and straightness (distance divided by displacement) plotted over pseudotime for each wild-type replicate. (**C**) Elbow plot used to choose the number of transition points. (**D**) Cell motion states mapped back onto the embryo. Compare to Fig 3d and e.

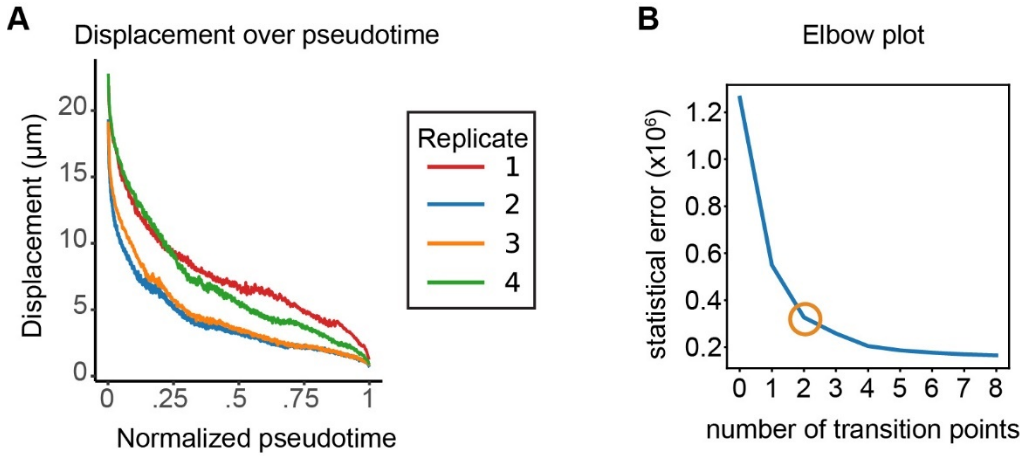

Fig S6. Cell motion segmentation information.

(**A**) Track displacement plotted over pseudotime for each replicate. (**B**) Elbow plot used to select the number of transition points.

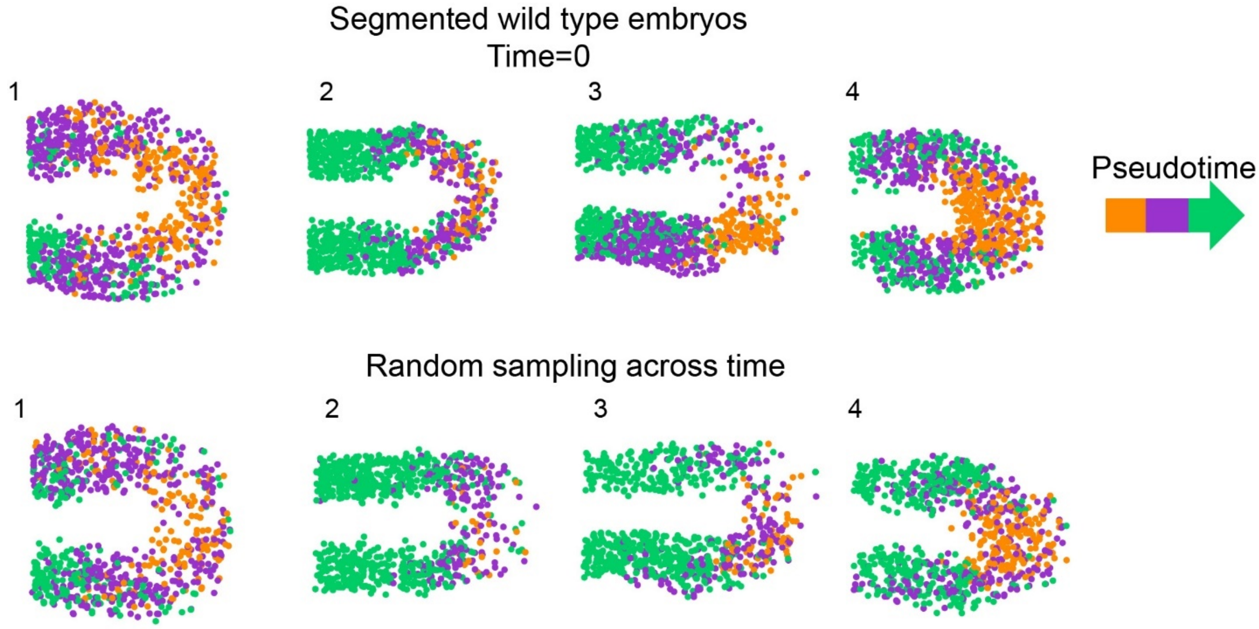

Fig S7. Pooled segmentation of wild type embryos.

One unified pseudotime was created and segmented for all replicates. Aside from replicate 1, this yields a similar result to segmenting each embryo individually.

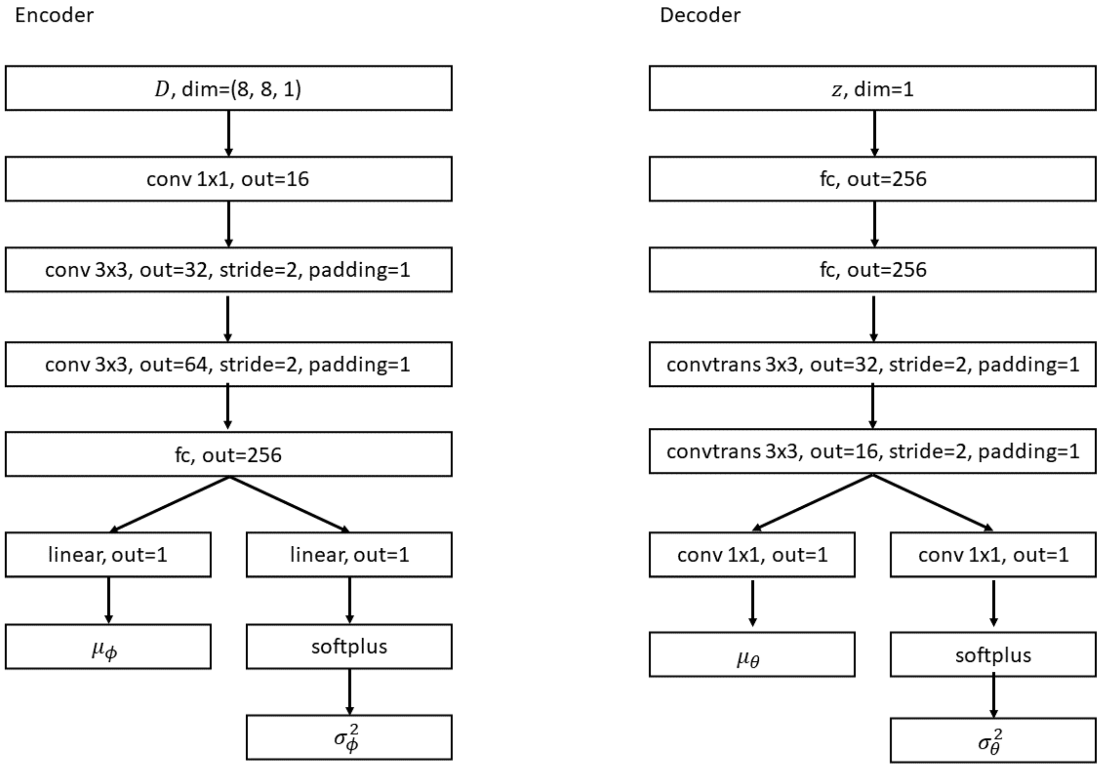

Fig S8. Architecture of VAE encoder and decoder networks.

conv and convtrans layers are implemented with Conv2d or ConvTransposed2d, BatchNorm2d and ReLU in PyTorch. fc layers are implemented with Linear, BatchNorm1d and ReLU in Pytorch.

Movie S1. Timelapse image of cell motion states.

Each dot marks a cell nucleus colored by cell motion state.
